# Leading new, deeper forms of collaborative cultures: Questions and pathways

**DOI:** 10.1007/s10833-021-09448-w

**Published:** 2022-01-11

**Authors:** Cecilia Azorín, Michael Fullan

**Affiliations:** 1 Faculty of Education, University of Murcia, Campus de Espinardo, 30100 Murcia, Spain; 2 498 St Clair Ave East, Toronto, M4T 1P7 Canada

**Keywords:** Leadership, Collaboration, Networking, Educational change, Innovation, Whole system transformation

## Abstract

The pandemic has made deeper problems more transparent and has stimulated many to realize that there may be an opportunity over the next period to pursue much needed innovations in learning. In this essay we describe the ways in which the pandemic has provided the conditions for new human development that joins two powerful forces: the pulsar model which elevates human potential with respect to student learning, and new, deeper forms of collaboration that have long eluded those interested in system change. In this article we show how ‘spirit work’ and collaboration can combine to develop schools systems that are essential for coping with the new post-pandemic conditions facing humanity. We also identify spinoff opportunities arising from the pandemic, and a corresponding pressure that could generate more widespread system improvement designed to improve learning for all, including advances in both equity and excellence.

## Introduction

The very fabric of education has been reshaped during the pandemic (Harris & Jones, 2020). COVID-19 has exposed the weaknesses of the learning culture of schools and has provided the opportunity for new developments. One area that has drawn particular interest is collaboration. This article examines new, deeper forms of collaboration enabled partly by the pandemic. Starting first with the evolution of collaborative cultures, a brief history is described below:

- *Collaboration’s failure to develop* The first slow and halting attempts at collaborative cultures started in the 1960s. There were the so-called ‘progressive movements’ that were full of promise, but lacked specificity, and failed to seek political support (Fullan, 2020).
- *Uneven development* There has been a period of partial development since the 1990s where ad hoc collaborative cultures were established, but again were not very deep or sustained (Hargreaves, 2019; Hargreaves & Fullan, 2012).
- *Landing in the collaboration arena* As we will show in the course of this article the experiences of the pandemic have provided the occasion for some schools to develop deeper forms of collaboration which may carry over into post-pandemic times (Fullan & Edwards, 2022; Pinchot & Fullan, 2021). This may provide the opportunity to make collaboration and system networks a fundamental feature of education improvement.

The pandemic conditions that have led to this new development have been enormously challenging but for some it is resulting in the potential to develop a more dynamic learning system. For others—in the absence of collaborative capacity it can result in worsening outcomes.

## The journey

The pandemic has not only challenged the foundations of education, but also of humanity itself. As Fullan (2021) has argued:

The COVID-19 pandemic has upended virtually every aspect of humanity as we know it, shaking current civilization to its foundation. Amidst the death and destruction is a disruption so fundamental that it loosens and discombobulates the system in a way that creates openings for transforming the status quo (p. 2).

COVID-19 turned the world and the education systems upside down producing an unprecedented disruption in recent educational history. The vast dimensions of the COVID-19 have produced what has been termed a ‘supernova’ effect, which in turn has led to a reimagination of what kind of new education might emerge after the pandemic (Azorín, 2020a). Education will not be the same as it was prior to COVID-19. UNESCO (2020) noted that “we cannot return to the world as it was before” (p. 6). These conditions present an opening for “a global reset of education” and “a chance to take a close look at aspects of education systems that we have taken for granted for far too long” (Robinson, 2020, p. 7).

The pandemic has revealed the fault lines in traditional educational systems, creating an opportunity to re-think the role of education in societal development (Fullan & Quinn, 2020). This opportunity may modernise education systems for the twenty-first century, enabling all children and young people to thrive in this fast-changing world (Arnove, 2020; Darling-Hammond et al., 2020; Fullan et al., 2020; Goodwin, 2020; Tesar, 2021).

Schools of the future need to define new models of education and develop collaborative learning cultures that prepare students from childhood to be supported by their peers, to solve problems together, and to network and exchange knowledge in an increasingly interconnected world. Fischer et al. (2020) noted that:

If the world of working and living relies on collaboration, creativity, definition and framing of problems and if it requires dealing with uncertainty, change and intelligence that is distributed across minds, cultures, disciplines, and tools—then education should foster competencies that prepare students for having meaningful and productive lives in such a world. Schools, however, have in many cases moved in the opposite direction (p. 244).

In the fight against the pandemic, collaboration and cooperation have been crucial for survival at all levels, including education. According to Oxfam (2021):

The coronavirus pandemic has widened pre-existing inequality gaps, but it has also brought our shared experience, vulnerability and interconnectedness into focus. Our health and resilience are inextricably linked to those of our neighbours, as is our survival in the face of other economic, political, social and climate crises. Cooperation and collaboration are not a choice, they are the only way to go (p. 44).

Collaborative cultures are presented as one major solution for the future of teaching and learning. The biggest question that many have is: “Where to start?” The rest of this article focuses on how those schools and regions that are interested but have made little progress to date can start fostering collaborative cultures. Some related questions are: How can schools be transformed into collaborative learning cultures? What are the first steps to be taken to initiate the shift towards collaboration? How can collaboration within and across schools be developed and extended?

A central part of the solution is what we call the *Pulsar Model of Educational Change* within the context of the pandemic—a metaphor that we have formulated to capture the powerful dynamics and new possibilities for post-pandemic transformation of learning. The ‘Pulsar Model’ includes an axis of three interactive forces that combine to create fundamental new systems in a given field. The three forces consist of a Copernican-like reversal that places students at the centre. Copernicus revealed that the earth, as with other planets revolved around the sun—not the opposite. Analagously, the pulsar model places the students at the center. This evolution towards student-centered learning has, of course been occurring for some time. Our point is that the upheaval of the pandemic may turn out to be the impetus that completes this development. Furthermore, this same transformation requires and stimulates deeper forms of collaboration to be successful.

Overall the new ‘innovation field’ may enable new cultures to evolve around the ‘guiding light’ that helps establish and maintain direction toward deeper forms of student learning. The model explains, among other aspects, the increase of collaborative networking that is taking place in the field of education.

Surprisingly, as we said above, although collaborative schools have received some attention since at least 1960 there has been little substantial progress in defining their presence and impact, or in demonstrating collaborative schools as force for transformative change. For example, Mehta and Datnow (2020) in a thematic issue of the American Journal of Education (AJE) put out a call for research papers that focused on changing ‘the grammar of schooling’—a concept that has been used to refer to longstanding cultures of how schools operated in their traditional daily patterns of interaction. Their work draws on the existing concept previously developed by Tyack and Tobin (1994), among others. Several of the editors, including one of us (Fullan, 2020), concluded that while innovations (for example involving collaborative cultures) appeared in these schools, they did not go very deep, nor did they last. In a major report on ‘Right drivers for whole system success’, Fullan (2021) similarly concluded that ‘social intelligence’—educators working together in innovative, focused ways to transform learning—was not well established in school systems.

Although progress has been made in terms of collaboration and networking in education, there are still many vague discussions at the international level (Azorín, 2020c; Hargreaves & Fullan, 2020). Azorín (2022) explores international evidence from North America, Latin America, and Western Europe, and concludes that educational reforms are being implemented to encourage a culture of cooperation and networking, which demonstrates that networking is spreading roots in the field of education. *Networking* supported by technology has grown exponentially, with the emergence of informal networks that have tried to fill the gaps in the educational system during the pandemic (Azorín, 2020a). Our argument is further buttressed by Fullan and Edwards (2022) who demonstrate how eight school districts in the US not only coped well with under the conditions of Covid but actually strengthened bonds within their systems. They combined ‘spirit work’ (cultivating caring for students as a deep and enduring commitment (the centrality of students) with the’science of collaboration’ (deep forms of working together tied to spirit work).

## Driving the pulsar model of educational change

Azorín (2020a) used the term ‘supernova’ to describe the impact that COVID-19 has had on education and argued that “like the lifecycle of a star, the educational journey of the previous decades has come to an end” (p. 381). This ‘supernova effect’ has brought with it the potential for an unprecedented pedagogical renewal and change that could give rise to the real-time rapid development of new approaches to education.

**Fig. 1:**
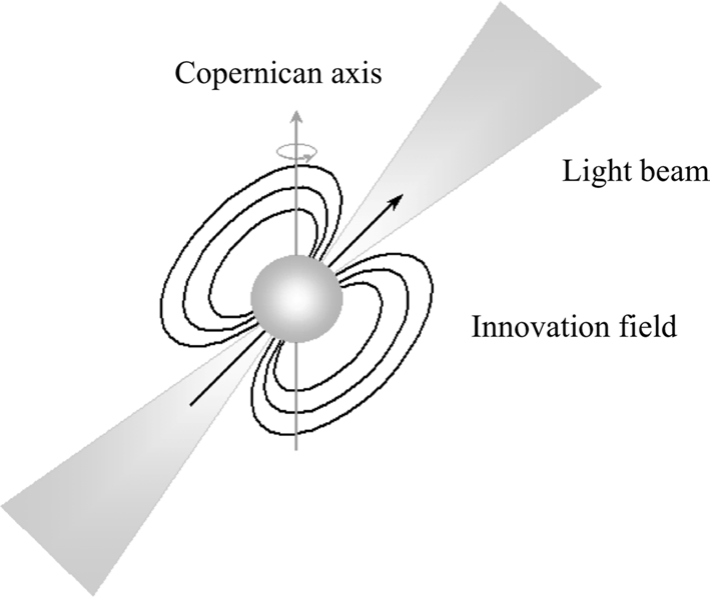
Pulsar model of educational change (image by Pogge, 2006)

The initial supernova drive has given way to what we call the pulsar model, where the change forces connect and interact thereby fostering processes of experimentation and innovation in education. Figure 1 shows the *Pulsar Model of Educational Change,* represented by a lighthouse (light beam) that illuminates the new educational pathways. In short, the Copernican axis represent the centrality of students; the light beam places collaboration at the center of action, and the innovation field concerns the pedagogical and collaborative developments essential for success.

### The Copernican axis

We made the case earlier that collaboration has not evolved to become a major force for system change. Here—in this essay—we are making the case that the pandemic has upended prior context to such a degree that many in the educational change field are considering what the new direction should be. In so doing, some are discovering the connection between moral purpose (spirit work) and forms of collaboration focused on such work.

The Copernican axis represents a radical, potentially rapid shift in education, which is associated with the disruption phase. As the pandemic has evolved, the Copernican turn in education has become more evident. The traditional teaching–learning process had placed children in school, but in effect did not place them at the centre. New collaboration requires greater joint or co-determination. With students for example, educators should teach their students to work effectively with each other. Shaw (2021) states that:

For many years, education has centred on the concept of individual students working on problems alone, memorising some random facts, and then regurgitating the information to the teacher in the form of a test. Teachers did not spend much time teaching students how to effectively use collaboration as a means of solving problems (p. 47).

Morever joint determination is expanded to include teachers and communities working together within and across (networks of) schools. The potential post-pandemic shift is to transform learning so that students are essential core partners in determining the nature of learning. Benefits to students in terms of motivation and belonging include a re-conceptualisation of teaching and learning as a collaborative process (Mercer-Mapstone et al., 2017).

Part of the shift should include transforming weak and superficial collaboration to deeper intra-school cultures of interaction nested in external networks, starting the Pulsar model with this phase that aspires to a change of practice and thinking. In the collaborative cultures that we are working with it is important to note that working together is about *the entire culture*: students, teachers, parents, administrators. These systems are led by ‘lead learners’ who model learning, but also cultivate learning in others—in the culture itself (see the eight district examples in Fullan & Edwards, 2022).

The pandemic has demonstrated the need to work towards a networked school, not only technologically speaking in terms of remote learning, but also from the perspective of collaboration, in the deepest and broadest sense of the word (Azorín, 2020b). The premise that “learning is confined to those with access to school buildings” (World Economic Forum, 2020, p. 7) has been made problematic with the pandemic. The perspective of students being drawn to the school as the centre of action is now being questioned. As we showed earlier, the new view is that students should be placed at the centre and the school must have a network to expand (technologically, collaboratively, organizationally) in order to ensure access to education and achievements for all. In essence, we use the Pulsar model as a metaphor that draws energy to the students as central, and points to focused collaboration as the main means of accomplishing fundamental change.

### The pedagogical renewal (the innovation field)

There was some pedagogical innovation prior to the pandemic, especially in our deep learning clusters. For example, one of us is involved in deep learning where innovations revolve around new pedagogical practices (students as partners in learning), expanding the learning environment, and leveraging technology for learning—all of these in the service of global competencies such as the 6Cs–character, citizenship, collaboration, communication, creativity, and critical thinking (Fullan et al., 2018; Quinn et al., 2020).

It is still an open question whether innovation will expand as a result of the pandemic. We do know that many districts deliberately shifted to focused innovation on the occasion of the disruption (such as the eight districts described in Fullan & Edwards, 2022). Within the pulsar effect, the magnetic field of innovation is attracting a pedagogical renewal that is based on the assumption that necessity is the mother of invention. It starts from the belief that a different education is possible. The pedagogical renewal that is underway is part of an innovative movement to promote accelerated educational change. It results in fundamental innovations such as the shift from traditional assessments toward performance-based assessment of the ‘backpack of skills’ in grades 5, 8, and 12 in Jefferson County Public Schools (Fullan & Edwards, 2022). These new models include ‘new purpose’ (to become ‘good at learning and good at life’); new competencies (character, citizenship, collaboration, communication, creativity & critical thinking); new learning design (pedagogy, partnerships, learning environment; leveraging digital); and new cultures and conditions (school, district or region, and central). All of these components are compatible with, indeed part and parcel of the Copernican axis.

### The lighthouse that guides education

The lighthouse concept, as we have said, puts the spotlight on innovations arising out of Covid that were enabled and even in some cases caused by the disruption and the opportunity it presented for those who were inspired and ready to take action. As a result of the movement of pedagogical renewal experienced in these times of crisis, and in order to assess what works in education, the contents summarized in the previous section reflect a new way of understanding learning that is emerging in contemporary education systems. The light beam symbolises a lighthouse that guides the new coordinates of the universe of education. It is part of the third stage of reinventing and reimaging both schools and education itself, which involves following the lights that illuminate educational change. It brings to the fore the unfinished business of inequity with solutions emerging that zero in on pedagogically based equity strategies.

Reflecting on the kind of education that the post-pandemic world needs, Hill et al. (2020) contends that it must address inequities, prioritise mental health and well-being in schools, decolonise teaching and learning, develop new models of education, build reciprocal relationships with the natural world, and recognise teachers as community leaders. The deep-learning model which *fuses* well-being and learning also serves as a guiding light. In this direction, according to Hatch (2020), the steps towards the future that schools can take to be more efficient, equitable, and effective are, among others:

- Make a commitment to address issues of equity, which implies building and sustaining partnerships across the health, education and economic sectors in order to mobilise resources in response to the crisis.
- Expand priorities to support all aspects of children’s development, by rethinking the main goals and content of schooling agenda and the prevalence of educational opportunities for all.
- Cut the curriculum in half, a premise based on the ‘less is more’ philosophy, promoting opportunities to connect and build positive relationships with peers and teachers. In line with this argument, Zhao (2021) called for education systems to think about the ‘building back better’ initiative and also to avoid the learning loss trap.
- Break down the barriers between learning inside and outside schools. Reinventing school space and time for learning inside and outside schools includes a change of focus ‘from getting children into schools, to taking advantage of the learning opportunities all around’ (p. 143).

## Navigating towards collaboration in education

There are several examples in the literature that describe collaboration and networking in education (Azorín, 2021; Griffiths et al., 2021; Nguyen and Ng, 2020; Paju et al., 2021; Pino-Yancovic & Ahumada, 2020; Rincón-Gallardo et al., 2019; Sherer et al., 2020). Although we have argued at the beginning that collaboration has not developed fully enough in previous decades, the pulsar that emerged in the context of the pandemic heralds a new era. There is now a timely opportunity to expand the use of networks along the lines we have been discussing. As Sinnema et al. (2021) put it, systems worldwide should “promote the use of collaborative networks to foster teacher learning and improve practice in the pursuit of educational change to address longstanding equity and achievement issues” (p. 1). In the last 60 years collaborative cultures appear primarily as *individual schools,* and in a few cases involve networks of school ranging in size from 10 to 150 schools, but there has been very little close examination of such networks (for one positive example see Rincón-Gallardo & Fullan, 2016).

It is no accident that more ambitious examples of collaboration with focus and depth are beginning to emerge such as Scotland’s new attempt at system-wide improvement. After a period of rejecting top-down change, in favor of a more laissez-faire approach Scotland around 2015 attempted to tighten up coordination of the entire system. The result is a major restructuring that divided the 32 Local Authorities in 6 Regional Improvement Collaboratives (RICS). The agency helping to coordinate the work is the Robert Owen Centre for Educational Change at the University of Glasgow. The Centre just produced its first major publication for which Fullan wrote the Foreword (Chapman & Ainscow, 2021). This initiative focusses on the right focus titled as Education equity: pathways to success. The research shows promising direction, but is in its early stages. Our main point is that this type of whole system strategy and research is likely to become more prominent because it arises naturally from the gaps and opportunities associated with the pandemic and its emerging aftermath. To be clear, the evolution has gone from collaborative schools, to collaborative networks of schools, to the current outstanding question: “How do schools and networks relate to ‘whole system improvement’? The adoption of collaborative cultures in education requires navigation through all levels within the system: policy, educational community, school leadership, and teacher level and beyond.

- Policy level: a global and local direction to move towards

During the pandemic, powerful networking and collaborative practices have emerged without policy decisions being imposed by the authorities (Sahlberg, 2020). Building a collaborative school culture involves more than a new policy programme or structure; it includes the assumption of values born within schools themselves (Ibrahim, 2020). However, if COVID-19 is used as a catalyst for educational change (Zhao, 2020), the first level can lay the foundations and provide some political support for the educational change to take place. The education authorities in different countries need to invest in networking and collaboration as key engines in the education policy agenda, and work to encourage the move of global and local efforts in this direction.

(b) Educational community level: the collective response

The second level seeks to mobilise the resources of the community and the environment to make them available for education. Using collective capacity is essential. Mortuza (2021) argues that at the educational community level:

More and more, people are realising that there has to be a concerted and collective response to stop our students from becoming castaway individuals marooned in lonely islands. Education is a collective effort, and if we do not invest in it, we will simply create further inequalities (p. 1).

Building a collaborative atmosphere entails engaging in reflection about whether promoting collaborative organisational culture is the responsibility of individual schools, or whether it extends beyond school walls. According to Bang et al. (2021) “schools can leverage partnerships with community-based organisations to support educators by sharing resources and organizing productive activities instead of relying on individual classroom teacher to do all of this work alone” (pp. 13–14).

(c) School leadership level: transforming agents for a collaborative culture

Educational leaders play a vital role in ensuring that schools are open to the development of collaborative cultures. In the context of COVID-19, educational leaders’ actions go beyond school limits and move towards a process of democratisation and openness in order to promote a culture of collaboration and ensure that networks generate the professional capacity to improve schools (Azorín et al., 2020; Azorín, 2020b; Fullan & Quinn, 2020; Harris et al., 2021).

(d) Teacher level and beyond: learning how to collaborate

Currently there is an increasing demand for teacher collaboration and professional learning networks that bring together teachers from different backgrounds for the co-construction of activities, mutual classroom observations, reducing isolation and exchange of experiences and resources, among many other opportunities (Schuster et al., 2021). At the same time, teachers should teach their students to be collaborative and network with one other as well, adopting cooperative work structures in the classroom and developing in their students a critical and practical thinking perspective. Student collaboration connected with teacher, school, and system development is part and parcel of the same fabric in the model we have presented in this paper. It is still at the early stages so we can’t be sure that it will expand. System change in education is notoriously fragile at the beginning stages.

## Conclusion

In historical perspective our argument makes the following case. From its origin as teaching as a lonely profession (‘behind the classroom door’), collaboration since the 1960s has made halting progress. Some strong collaborative school cultures were established over the decades, but they were limited in three ways: they were in the minority; were mostly intra-school with a smattering of school districts; and they did not become an established part of a new culture. Over the past decade we have begun to see examples of networks of schools, but these too did not represent system change. Recently (mostly in the past two or three years) there is a new and powerful surge in collaboration arising from the combination two forces: first, the growing evidence that traditional school systems have been seen as ineffective for the majority of students having lost their sense of purpose (see Fullan, 2021), and second, that the pandemic has exposed the weakness of the school system, and serendipitously increased the interest in innovation and system reform as we enter the post-pandemic period (Fullan & Edwards, 2022).

Prior to COVID-19, there was consensus on the need to prepare future generations in environments of collaboration (Azorín, 2022), but it did not materialize in practice. The pandemic has accelerated networking in education as a powerful tool for innovation. Collaboration is needed and the pandemic made this need greater. “Teaching today is a collaborative and social profession” which implies “moving ideas, knowledge, and teaching practices around in professional communities and networks of shared professional learning” (Hargreaves, 2021, p. 142). We see these developments emerging (and, indeed are part of networks ourselves working on this very agenda). We predict that this recent trend will take off in the coming years.

The Pulsar model with its three core dimensions captures this direction: (1) the flipping of the axis from teacher-centered to student-centered; (2) the field that generates pedagogical innovations; and (3) the lighthouse that illuminates well-being and learning as the goal of humanity for surviving and thriving in an ever complex society. Accordingly, we recommend that the field of education and learning *not* simply think of extending past knowledge of collaborative cultures, but rather take a ‘system perspective’. By so doing collaboration, and whole system success including equity for all could make major advances in the current decade.
